# T67. NEUROLOGICAL SOFT SIGNS IN THE COURSE OF SCHIZOPHRENIA: A LONGITUDINAL ANALYSIS IN CHRONIC SCHIZOPHRENIA

**DOI:** 10.1093/schbul/sby016.343

**Published:** 2018-04-01

**Authors:** Christina Herold, Céline Duval, Marc Lässer, Ulrich Seidl Seidl, Dusan Hirjak, Philipp Thomann, Johannes Schröder

**Affiliations:** 1 University of Heidelberg; 2 Rehabilitation Hospital Zihlschlacht; 3 Center for Mental Health, Klinikum Stuttgart

## Abstract

**Background:**

Neurological soft signs (NSS) are minor (‘soft’) neurological abnormalities in sensory and motor performance, which are frequently reported in patients with schizophrenia at any stage of their illness. It has been demonstrated that NSS vary in the clinical course of the disorder: Longitudinally NSS seem to decrease in parallel with remission of psychopathological symptoms, an effect which mainly applies to patients with a remitting course. However, these findings are mainly based on patients with a first episode of the disorder and the course of NSS in patients with chronic schizophrenia and persisting symptoms is rather unknown.

**Methods:**

Therefore, we investigated 21 patients with chronic schizophrenia (duration of illness: 22.8 years ± 11.5) twice with a follow-up time interval of 7 years. Baseline and endpoint NSS scores were evaluated by the Heidelberg Scale, established instruments were used to rate psychopathological symptoms and neuropsychological performance.

**Results:**

Results show a significant increase of the NSS subscales “motor coordination” and “integrative functions” with stable positive and negative symptoms, including apathy, as well as chlorpromazine equivalents. Along with this, neuropsychological parameters as verbal memory, verbal fluency and cognitive flexibility deteriorate significantly. Regression analyses show that the TMT B performance/cognitive flexibility and the SANS global score/negative symptoms at baseline are strong predictors for NSS increase at follow-up.

**Discussion:**

These results illustrate a significant aggravation of NSS in patients with chronic schizophrenia over time, while psychopathological symptoms remain stable. In addition, cognitive performance is deteriorating as well, with cognitive flexibility together with negative symptoms as strongest predictors for NSS changes.

